# Labelling HaloTag Fusion Proteins with HaloTag Ligand in Living Cells

**DOI:** 10.21769/BioProtoc.2526

**Published:** 2017-09-05

**Authors:** Huy Nguyen Duc, Xiaojun Ren

**Affiliations:** Department of Chemistry, University of Colorado Denver, Denver, CO, USA

**Keywords:** HaloTag, Live-cell single-molecule imaging, Polycomb, Cbx, Epigenetics, Janelia Fluor^™^ dye

## Abstract

HaloTag has been widely used to label proteins *in vitro* and *in vivo* (Los *et al*., 2008). In this protocol, we describe labelling HaloTag-Cbx fusion proteins by HaloTag ligands for live-cell single-molecule imaging (Zhen *et al*., 2016).

## Background

Molecular processes of living organisms are intrinsically dynamics. Direct observation of the molecular processes in living cells is critical for quantitatively understanding of how biological systems function. Recent advances in fluorescence microscopy and fluorescent labelling enable to visualize trajectories of individually single molecules in living cells, providing insights about dynamic interactions and assemblies of biological molecules (Kusumi *et al*., 2014; Liu *et al*., 2015; Tatavosian *et al*., 2015; Cuvier and Fierz, 2017). Specific labelling of biomolecules with fluorophores is the key for fluorescence single-molecule imaging. HaloTag is self-labeling tag proteins that can be coupled to synthetic dyes in living cells (Los *et al*., 2008). The reaction occurs rapidly in living cells and the formed covalent bond is specific and irreversible. This technique has been utilized to study the genetic information flow *in vivo*, and to measure the kinetic of gene regulation in living mammalian cells (Liu *et al*., 2015; Zheng and Lavis, 2017). Janelia Fluor^™^ dyes, such as Janelia Fluor^™^ 549 (JF_549_), are bright and photostable fluorescent HaloTag ligands (Grimm *et al*., 2015). This protocol describes how to label HaloTag-Cbx proteins with JF_549_ for live-cell single-molecule imaging, which was developed in the recent publication (Zhen *et al*., 2016).

## Materials and Reagents

1. Pipette tips (BioExpress, catalog number: P-1236-200)
  Manufacturer: Biotix, catalog number: P-1236-200CS.
2. 35 mm glass bottom dish made in the laboratory (see Video 1 for making glass-bottom dishes)
3. Cell lines used in protocol: mouse embryonic stem cells and HEK293T cells
4. Janelia Fluor 549 dye (JF_549_) provided by Dr. Luke D. Lavis (Janelia Research Campus, Howard Hughes Medical Institute)
5. Trypsin-EDTA (Thermo Fisher Scientific, Gibco^™^, catalog number: 25300054)
6. Phosphate-buffered saline (PBS) (Sigma-Aldrich, catalog number: D8537-500ML)
7. Gelatin (Sigma-Aldrich, catalog number: G1890-100G)
8. DMEM (Sigma-Aldrich, catalog number: D5796)
9. Fetal bovine serum (FBS) (Sigma-Aldrich, catalog number: F0926)
10. Glutamine (Thermo Fisher Scientific, Gibco^™^, catalog number: 25030081)
11. Penicillin-streptomycin (Thermo Fisher Scientific, Gibco^™^, catalog number: 15140122)
12. β-Mercaptoethanol (Thermo Fisher Scientific, Gibco^™^, catalog number: 21985023)
13. Non-essential amino acids (Thermo Fisher Scientific, catalog number: 1114050)
14. Leukemia inhibitor factor (LIF, made in the laboratory)
15. FluoroBrite DMEM (Thermo Fisher Scientific, Gibco^™^, catalog number: A1896701)
16. ES cell medium (see Recipes)
17. Live-cell imaging medium (see Recipes)

## Equipment

1. Single channel pipette (BioExpress, Kaysville, USA)
2. Heater controller (Warner Instruments, catalog number: TC-324)
3. Microscope (Manual Microscopy) (ZEISS, model: Axio Observer D1)
4. Alpha Plan-Apochromatic 100/1.46 NA Oil-immersion Objective (ZEISS, Germany)
5. Evolve 512 × 512 EMCCD camera (Photometrics, Tucson, USA)
6. Solid state laser (Intelligent Imaging Innovations, 3i LaserStack with Fiber)

## Software

1. Slidebook 6.0 software (Intelligent Imaging Innovations, Denver, Colorado)
2. MATLAB R2015a (8.5.0.197613) (MathWorks, Natlilck, USA)
3. U-track 2.0 (Danusar Lab, UT South Western Medical Center, Dallas, USA)

## Procedure

1. Trypsinize 70–90% confluent cells (mouse embryonic stem cells or HEK293 cells) of 100 mm plate stably expressing HaloTag-Cbx proteins (we recommend 0.6 ml of 0.05% trypsin-EDTA [1x] for 60 mm plate and 1.5 ml for 100 mm plate).
2. Seed 20% of cells to 35 mm glass-bottom dish coated by Gelatin overnight (Note 1) (see Video 2 for gelatinization).
3. Following by overnight culture, the final confluency of the cells before was between 80–90%. Several concentrations (5 nM, 15 nM, and 30 nM) of JF_549_ are used to incubate with cells for 15 min at 37 °C in 5% CO_2_ (Notes 2 and 3) (see Video 3 for adding the JF_549_ dye).
4. Gently wash cells with ES medium (see Recipes) once and incubate in ES medium for 30 min at 37 °C and 5% CO_2_ (see Video 4 for washing cells).
5. Replace ES medium with live-cell imaging medium (see Recipes) (see Video 5 for adding live-cell imaging medium).
6. Maintain 37 °C conditions during imaging by using a heater controller. Each plate should be imaged for the maximum of 1.5 h after placing on the microscope (see Video 6 for placing dishes on objective).
7. The number of individual fluorescent spot per nucleus should be between 10–50 spots, controlled by adjusting the JF_549_ dye concentrations (Notes 4 and 5) (see Video 7 for single-molecule imaging).
8. Movies are then uploaded to u-track 2.0. Each cell is cropped from the larger movie. Cropped movies are processed (Note 6).

## Data analysis

1. Our data were analyzed using MATLAB with u-track 2.0 plug-in, detail guide can be found at http://www.utsouthwestern.edu/labs/danuser/software/ (the software and pdf file guide are included in the download).
2. Representative images and movies can be found in Zhen *et al*., 2016.

## Recipes

1. ES cell medium
  DMEM
  15% FBS
  2 mM glutamine
  100 U/ml penicillin-streptomycin
  0.1 mM β-mercaptoethanol
  1,000 U/ml LIF
  0.1 mM non-essential amino acids
2. Live-cell imaging medium
  FluoroBrite DMEM supplemented with 15% FBS
  2 mM glutamine
  100 U/ml penicillin-streptomycin
  0.1 mM β-mercaptoethanol
  1,000 U/ml LIF
  0.1 mM non-essential amino acids

## Figures and Tables

**Video 1:**
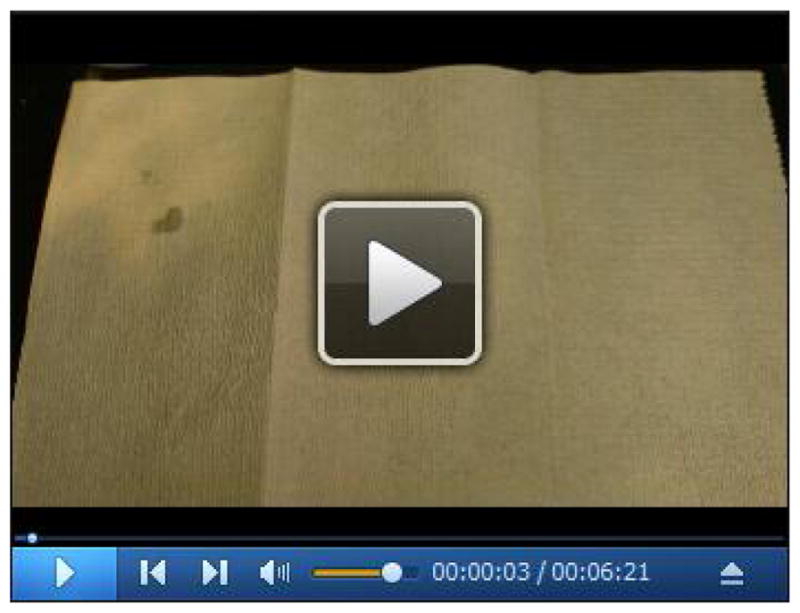
Making glass-bottom dishes The video elaborates how to make glass-bottom dishes for live-cell single-molecule imaging.

**Video 2:**
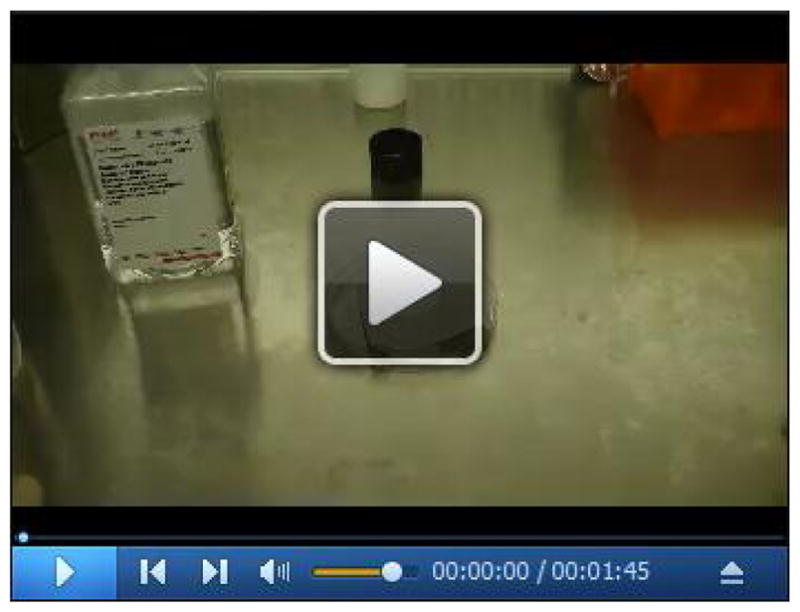
Gelatinization of glass-bottom dishes The video describes how to gelatinize glass-bottom dishes before seeding cells.

**Video 3:**
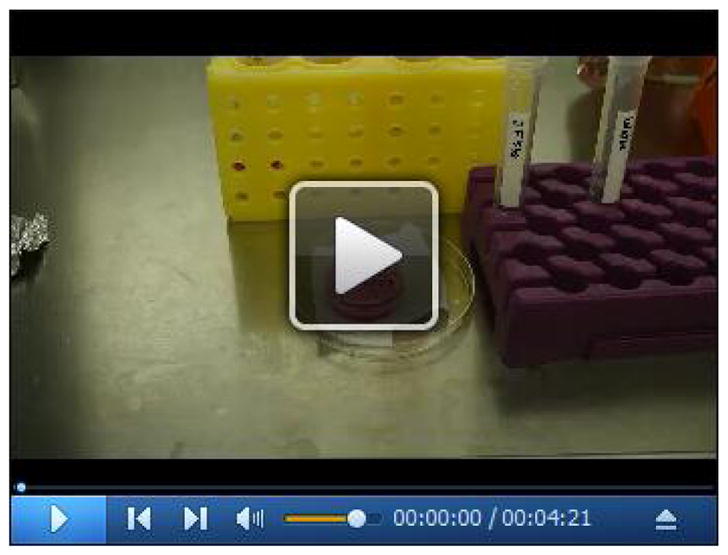
Adding JF_549_ dyes to cells

**Video 4:**
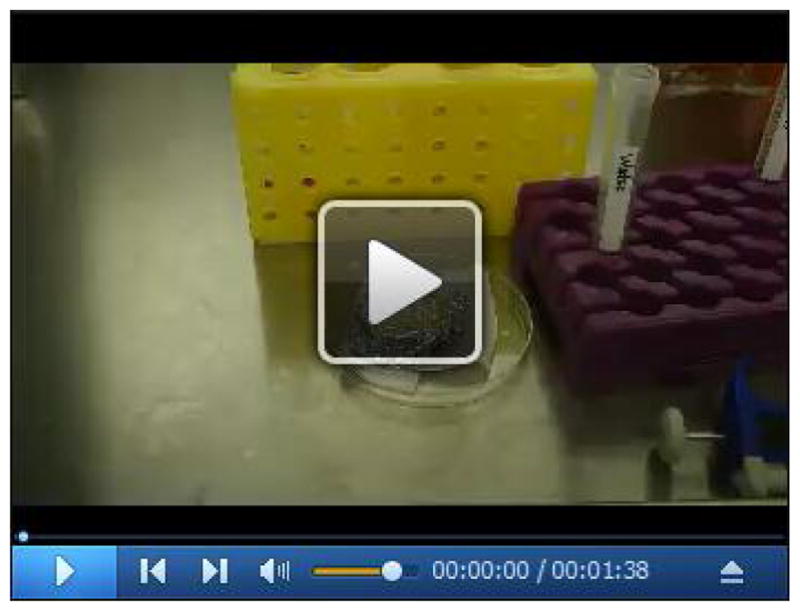
Washing cells with ES cell medium

**Video 5:**
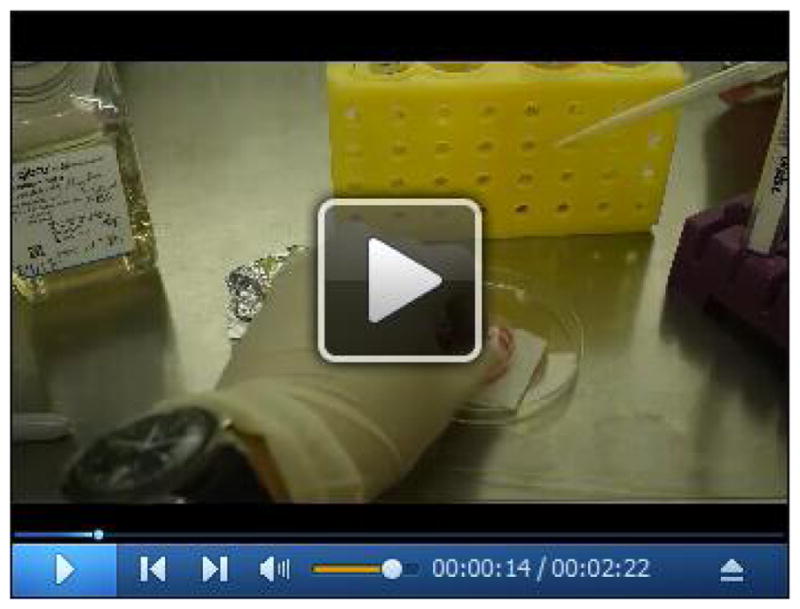
Replacing ES cell medium with live-cell imaging medium

**Video 6:**
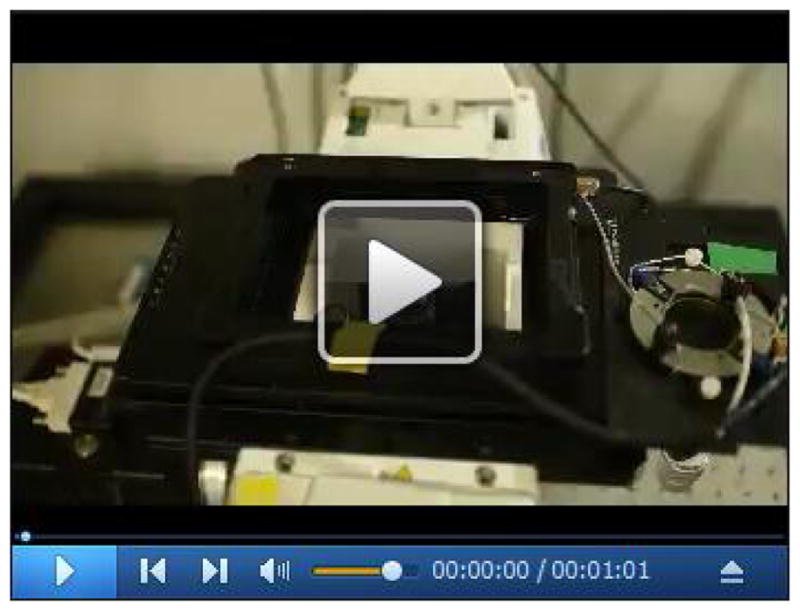
Placing dishes on objective

**Video 7:**
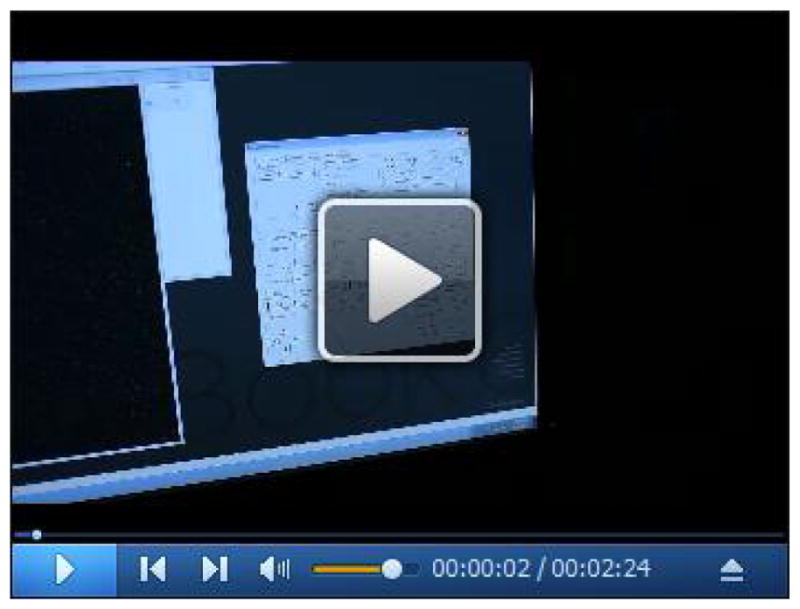
Single-molecule imaging The video describes how to image individual HaloTag-Cbx proteins within living cells.

## Footnotes

1 Imaging dishes should be gelatinized overnight.

2 We recommend using pipettes, not suction, when removing medium from imaging dishes.

3 When add medium to the cells in imaging dishes, we recommend tilting plate at an angle, slowly add medium to the lower edge and let the medium reach the cells slowly.

4 The TIRF angle used is unique for individual cells and should be adjusted to achieve the best possible movies.

5 To obtain representative movies, the focus of the movie should be at the middle layer of the nucleus. This can be recognized by the deeper black area (this can be achieved by adjusting the angle). Bright field can be used to check the nucleus area.

6 In u-track 2.0, multiple movies can be loaded and processed. After being processed, each movie should be checked to ensure that cells should be no drift and rotation.
